# Development of Nonbacterial Thrombotic Endocarditis While on Systemic Anticoagulation in Pancreatic Cancer: A Case Report

**DOI:** 10.7759/cureus.10967

**Published:** 2020-10-15

**Authors:** Jiasheng Wang, Natasha Monga, Prashanth Mopala, Muhammad Husnain

**Affiliations:** 1 Department of Internal Medicine, MetroHealth Medical Center, Cleveland, USA; 2 Department of Radiology, MetroHealth Medical Center, Cleveland, USA; 3 Department of Cardiology, MetroHealth Medical Center, Cleveland, USA; 4 Department of Medicine, MetroHealth Medical Center, Cleveland, USA

**Keywords:** nonbacterial thrombotic endocarditis, pancreatic cancer, anticoagulation

## Abstract

Nonbacterial thromboembolic endocarditis (NBTE), or marantic endocarditis, is a rare complication associated with advanced cancer. Enoxaparin or unfractionated heparin is considered the standard treatment for NBTE. In this case report, we describe a 59-year-old female with metastatic pancreatic cancer who presented with embolic stroke and was found to have new NBTE of the mitral valve while she was receiving the therapeutic dose of enoxaparin. Of note, her recent echocardiogram one week ago was negative for mitral valve vegetations. Our case emphasized that for patients with advanced cancer presenting with stroke, the diagnosis of NBTE should be entertained even for those on systemic anticoagulation.

## Introduction

Marantic or nonbacterial thromboembolic endocarditis (NBTE) is a rare event seen in patients with advanced malignancy [1]. The vegetations of NBTE are commonly noninflammatory platelet-fibrin deposits usually located on aortic and mitral valves. The treatment for NBTE involves systemic anticoagulation with a therapeutic dose of low molecular weight heparin (LMWH) or unfractionated heparin (UFH). The development of new NBTE and embolic stroke while on LMWH has not been reported.

## Case presentation

**Figure 1:**
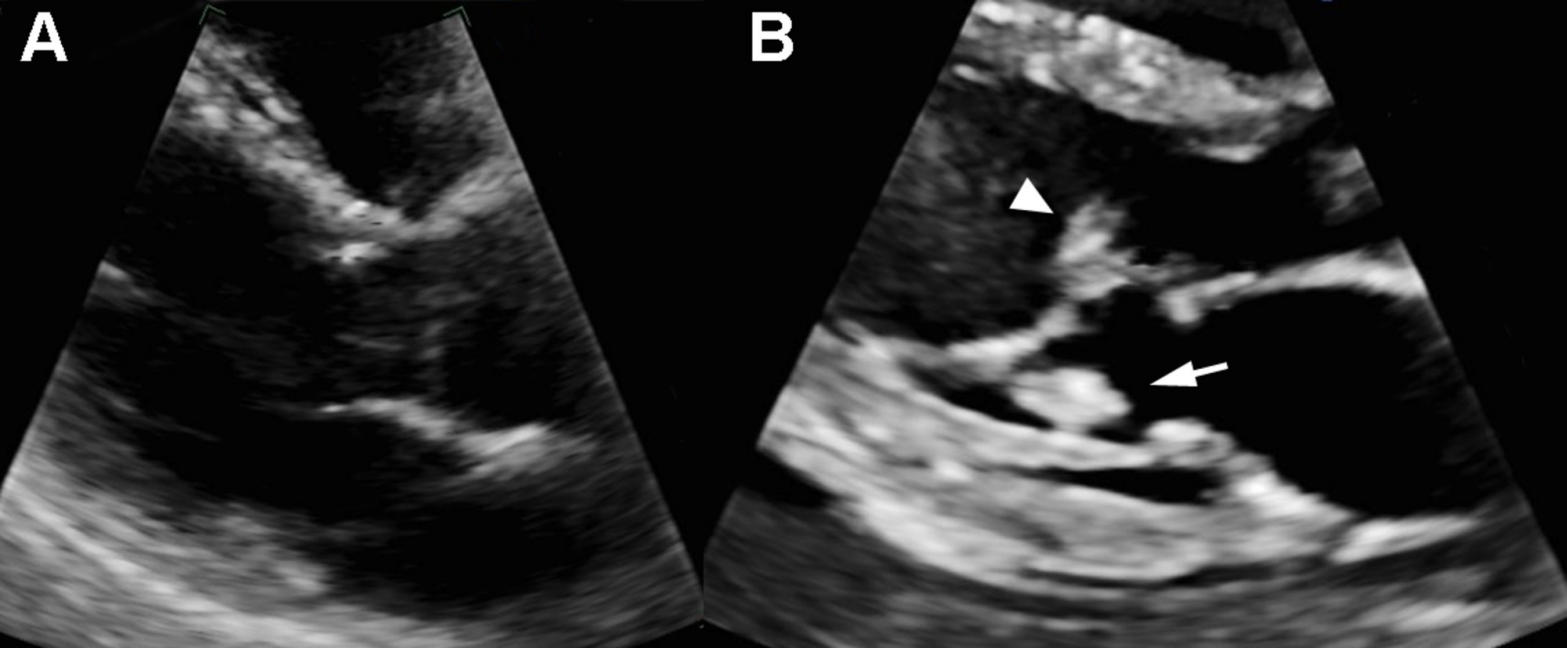
Echocardiogram two weeks prior and on the day of admission Two weeks prior to the current presentation, no obvious vegetations were identified on echocardiogram (A). A repeat echocardiogram at this admission (B) demonstrated two vegetations attached to the anterior leaflet chordae (arrowhead) and the posterior leaflet chordae (arrow) of the mitral valve, respectively.

A 59-year-old female was admitted to the hospital for shortness of breath and found to have bilateral pulmonary emboli diagnosed by computed tomography pulmonary angiogram. Incidentally noted were numerous ill-defined hypodense hepatic lesions with subsequent percutaneous liver biopsy consistent with metastatic pancreatic adenocarcinoma. A transthoracic echocardiogram (TTE) was also performed at that time (Figure 1), which showed unremarkable valves (Figure 1A). The patient was discharged home on the therapeutic dose of enoxaparin (1 mg/kg administered subcutaneously twice a day).

**Figure 2:**
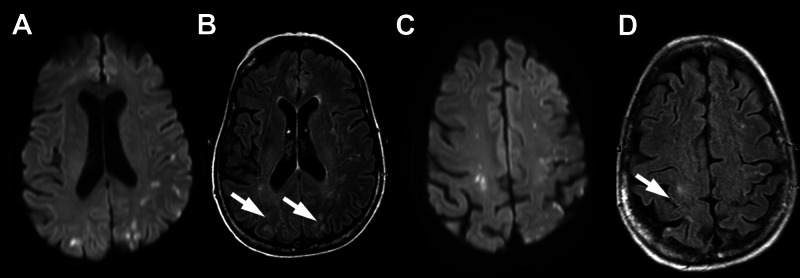
Brain MRI on the day of admission Diffusion-weighted sequences (A and C) demonstrate numerous cortical and subcortical foci of restricted diffusion in frontal and parietal lobes. On the FLAIR sequences (arrows B and D), these foci are only mildly hyperintense, consistent with acute embolic infarcts involving the anterior circulation. MRI: magnetic resonance imaging; FLAIR: fluid-attenuated inversion recovery

Two weeks later, the patient presented from the oncology clinic with acute confusion for one day. Of note, no treatment for pancreatic cancer has been started. Laboratory tests showed normal renal function, normal fibrinogen level, and normal platelet counts; activated partial thromboplastin time was normal, but prothrombin time was slightly prolonged (15 seconds, normal 9.7­ to 12.9 seconds). Two sets of blood cultures were obtained, which returned negative. Unenhanced magnetic resonance imaging of the brain demonstrated numerous foci of restricted diffusion bilaterally, consistent with microemboli (Figure 2).

A limited TTE showed two new masses, measuring 1.2×0.7cm and 1.1×0.9cm, attached to the mitral valve anterior leaflet chordae and the posterior leaflet chordae, respectively (Figure 1B). Of note, a family member reported that the patient had been administering enoxaparin consistently. Enoxaparin was continued in the hospital. However, her mental status continued to decline, and she became comatose in 24 hours. The family elected comfort care, and the patient passed away after four days.

## Discussion

In this case report, we described a patient with recently diagnosed metastatic pancreatic cancer who developed NBTE that led to the formation of cerebral microemboli while on the therapeutic dose of LMWH for pulmonary embolism. The mechanisms of ischemic stroke in cancer patients are often undetermined. However, NBTE seems to be a leading cause as evidenced by an autopsy study [2]. Diagnosing NBTE with echocardiogram is often difficult due to the small sizes of vegetations. Nonetheless, they are extremely thrombogenic and can dislodge easily due to loose attachment to undamaged cardiac valves. Moreover, several features in our patient, such as mitral valve involvement, a size larger than 10 mm, and more than one vegetations, are associated with a higher risk of embolism [3]. LMWH or UFH is the standard treatment for NBTE [4]. The evidence of anticoagulation comes from observational studies, where improved neurological symptoms were observed when anticoagulation was initiated [5]. For patients with late-stage pancreatic cancer, a new occurrence of NBTE while on LWMH has not been reported. However, the literature review showed recurrent embolic stroke while on systemic anticoagulation was common in this population (Table 1).

**Table 1:** Literature review of nonbacterial thrombotic endocarditis in patients with pancreatic cancer NBTE, nonbacterial thrombotic endocarditis. ATE, arterial thromboembolism. M, male. F, female. MV, mitral valve. AV, aortic valve. AIS, acute ischemic stroke. N/A, not available. TIA, transient ischemic attack. MI, myocardial infarction. UFH, unfractionated heparin. LWMH, low-molecular-weight heparin.

Study	Patient age (years), sex	Cancer stage	Location of NBTE	ATE presentation	Size of NBTE	Treatment	Neurological outcome
Jameson et al. [6]	64, M	IV	Anterior leaflet of MV	AIS	N/A	Warfarin	Recurrent AIS
	61, M	IV	Across intra-atrial septum	AIS	N/A	Warfarin	Recurrent TIA
Sia et al. [7]	54, F	Locally advanced	AV	AIS, MI, limb ischemia	7×4mm	None	Died
Smeglin et al. [8]	43, F	IV	AV	Limb emboli	N/A	UFH	N/A
Piovanelli et al. [9]	48, F	N/A	AV	AIS	N/A	UFH	Recurrent AIS
Starobinska et al. [10]	66, M	Locally advanced	Posterior leaflet MV	AIS	11×7mm	Systemic anticoagulation	Stable
Chen et al. [11]	81, F	IV	MV	AIS, MI	6×8mm, 5×5mm	None	Died
Mantovani et al. [12]	65, F	N/A	AV, MV	None	N/A	LWMH	N/A
Takeshita et al. [13]	65, F	IV	AV	AIS	N/A	UFH	Recurrent AIS

Therefore, the occurrence of new NBTE in our patient likely reflects the hypercoagulability associated with pancreatic cancer. However, the development of antiphospholipid antibody or medication noncompliance could not be excluded. For patients with new NBTE while on anticoagulation, little evidence is available for alternative treatments. However, the use of direct oral anticoagulants (DOAC) should be cautioned, as Mantovani et al. [12] reported a patient who developed NBTE while on rivaroxaban, but vegetations disappeared after DOAC was switched to LWMH.

## Conclusions

In conclusion, for patients with advanced cancer presenting with ischemic stroke, the diagnosis of NBTE should be entertained even for those on systemic anticoagulation. 
